# Early Predictive Markers and Histopathological Response to Neoadjuvant Endocrine Therapy in Postmenopausal Patients with HR+/HER2− Early Breast Cancer

**DOI:** 10.3390/cancers17142319

**Published:** 2025-07-12

**Authors:** Aleksandra Konieczna, Magdalena Rosinska

**Affiliations:** 1 Department of Breast Cancer and Reconstructive Surgery, Maria Sklodowska-Curie National Research Institute of Oncology, 02-781 Warsaw, Poland; 2 Digital Medicine Centre, Maria Sklodowska-Curie National Research Institute of Oncology, 02-781 Warsaw, Poland; magdalena.rosinska@nio.gov.pl

**Keywords:** neoadjuvant endocrine therapy, HR-positive breast cancer, Ki-67, progesterone receptor (PgR), biomarkers

## Abstract

**Purpose**: Neoadjuvant endocrine therapy (NET) represents a valuable treatment option for hormone receptor-positive (HR+)/HER2-negative breast cancer, particularly in postmenopausal women. This study aimed to evaluate the clinical and histopathological efficacy of NET and to explore early and late changes in *Ki-67* and progesterone receptor (PgR) expression as indicators of endocrine response. **Methods**: A prospective cohort of 127 postmenopausal patients with stage cT1–4N0–3M0 HR+/HER2− breast cancer was enrolled between 2019 and 2021. Patients received NET (mostly letrozole) for a mean of 7.7 months. In 80 cases, a second core biopsy was performed after four weeks. Tumor size, histological grade, and biomarkers (*Ki-67*, PgR) were assessed pre- and post-treatment. **Results**: NET led to a significant reduction in tumor size, with median shrinkage of 47.0% (from 32.0 mm to 17.0 mm, *p* < 0.0001). Breast-conserving surgery (BCS) was performed in 52.2% of patients and lymph node negativity (pN0) was observed in 50.4%. Median Ki-67 decreased from 20.0% at baseline to 5.0% after four weeks (*p* < 0.0001) and remained low in surgical specimens (median 5.0%, *p* < 0.0001). In 33.3% of patients, *Ki-67* dropped below 2.7%, and 67.0% showed a concordant decrease in both *Ki-67* and PgR. PgR expression declined significantly during treatment (*p* < 0.0001). HER2 status conversion was noted in 6.4% of patients during treatment. Pathological complete response (pCR) occurred in 3.5%, while minimal or moderate residual disease (RCB I–II) was identified in 71.3% of cases. **Conclusions**: NET effectively reduced tumor burden and histological aggressiveness, enabling higher rates of BCS. Early reduction in *Ki-67* and PgR may serve as surrogate markers of endocrine responsiveness, supporting their use for treatment stratification and monitoring during NET in HR+/HER2− breast cancer.

## Simple Summary

Neoadjuvant endocrine therapy (NET) is a treatment option for postmenopausal patients with hormone receptor-positive (HR+)/HER2-negative early breast cancer, aimed at shrinking tumors before surgery. In this prospective study, we evaluated the effectiveness of NET with letrozole and explored early changes in tumor biology, including the proliferation marker *Ki-67* and progesterone receptor (PgR) expression. We found that NET significantly reduced tumor size and histological aggressiveness. Early declines in *Ki-67* and PgR were associated with favorable biological response and could help predict which patients benefit most from this therapy. Our findings support the use of dynamic biomarker monitoring to personalize treatment and improve outcomes in HR+/HER2− breast cancer.

## 1. Introduction

Systemic treatment in breast cancer can be administered either before or after surgery and includes chemotherapy, endocrine therapy, immunotherapy and molecularly targeted agents [1]. Neoadjuvant systemic treatment aims to reduce tumor burden to facilitate breast-conserving surgery or allow radical resection of initially inoperable tumors. Additionally, it offers a unique opportunity to evaluate early treatment response and identify predictive biomarkers [2].

Hormone receptor-positive, **HER2-negative (HR+/HER2−)** breast cancer is the most common subtype among postmenopausal women [3]. These patients often present with comorbidities that limit the use of cytotoxic chemotherapy. In this context, neoadjuvant endocrine therapy (NET) is an attractive, well-tolerated alternative [4]. Despite its demonstrated efficacy and low toxicity, NET remains underutilized, even in patients with **luminal** breast cancer, where it may be most beneficial. Clinical trials have confirmed its effectiveness across a variety of patient populations, yet robust guidelines for patient selection and monitoring of therapeutic response remain lacking [5].

Current molecular tools proposed for guiding NET include hormone receptor levels, *Ki-67* proliferation index, the preoperative endocrine prognostic index (PEPI) and multigene assays [6]. Among these, *Ki-67* has received particular attention as a potential early marker of endocrine sensitivity. Its dynamic change during treatment may reflect biological response to therapy and support risk-adapted management [7]. Similarly, downregulation of *progesterone receptor (PgR)* expression during NET may reflect altered hormone signaling and has been proposed as an additional biomarker of response [8].

In this prospective study, we aimed to evaluate clinical, biological, and histopathological responses to NET in a cohort of postmenopausal patients with HR+/HER2− early-stage breast cancer. Special attention was given to early changes in *Ki-67* and *PgR* expression and their association with treatment outcomes, including tumor downstaging, response classification, and surgical conversion rates.

## 2. Materials and Methods

### 2.1. Study Design and Patient Population

This was a prospective study evaluating the clinical, biological, and histopathological response to neoadjuvant endocrine therapy (NET) in postmenopausal patients with *hormone receptor-positive (HR+), HER2-negative (HER2−)* early breast cancer, with particular emphasis on the assessment of *Ki-67* as a dynamic biomarker. Between 2019 and 2021, 127 patients with clinical stage cT1–4, N0–3, M0 HR+/HER2− breast cancer initiated preoperative endocrine treatment at the Department of Breast Cancer and Reconstructive Surgery, Maria Sklodowska-Curie *National Research Institute of Oncology* in Warsaw, Poland (Table 1).

**Figure 1:**
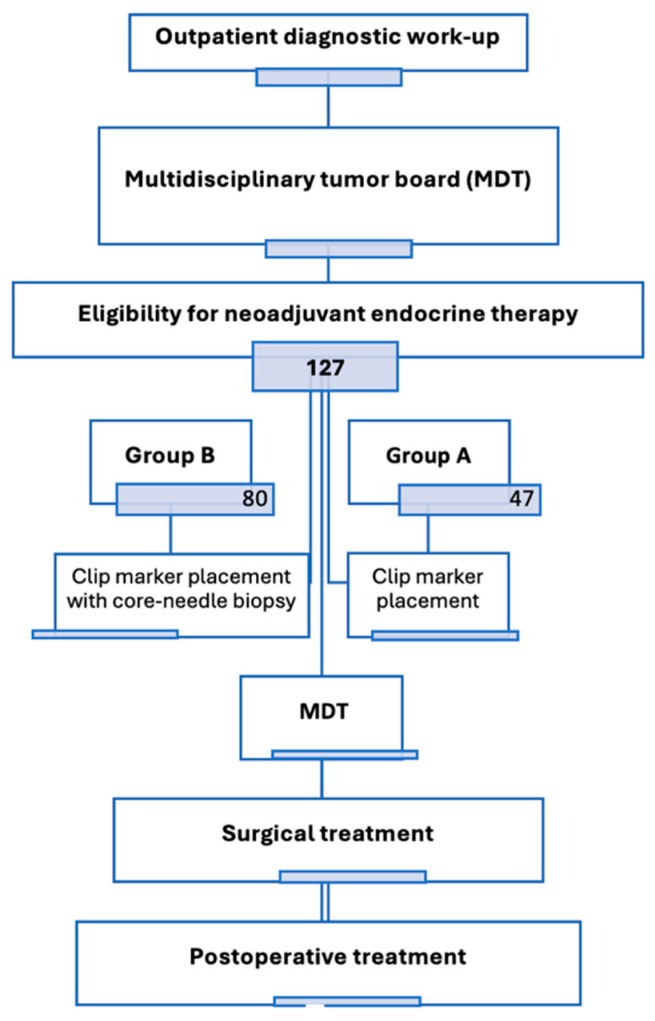
Study flowchart: patient allocation, procedures, and treatment sequence.

Patients were initially diagnosed at the institute’s outpatient clinic. Following diagnosis, all cases were reviewed by a multidisciplinary tumor board (MDT), which confirmed eligibility for NET according to institutional protocols (Figure 1). Primary diagnostic procedures included core needle biopsy of the breast tumor and clinically suspicious lymph nodes. Histopathological evaluation encompassed tumor subtype, histological grade, estrogen and progesterone receptor status, and *HER2* and *Ki-67* expression.

A subset of 80 patients (Group B) provided additional informed consent for a second core needle biopsy approximately 4 weeks after treatment initiation, performed during standard clip marker placement. This procedure did not require any extra intervention beyond standard clinical care. The remaining 47 patients (Group A) were treated according to routine clinical practice without interim biopsy. All patients were monitored every 2–3 months with physical examination and additional tests as required. Upon completion of NET, all patients were re-evaluated by the MDT and referred for surgery.

Ethical approval for this study was obtained from the Bioethics Committee of the National Institute of Oncology, approval number 22/2019. All procedures involving human participants were conducted in accordance with the institutional and national ethical standards and the 1964 Helsinki Declaration and its later amendments. Written informed consent was obtained from all participants.

### 2.2. Treatment Regimen

**Figure 2:**
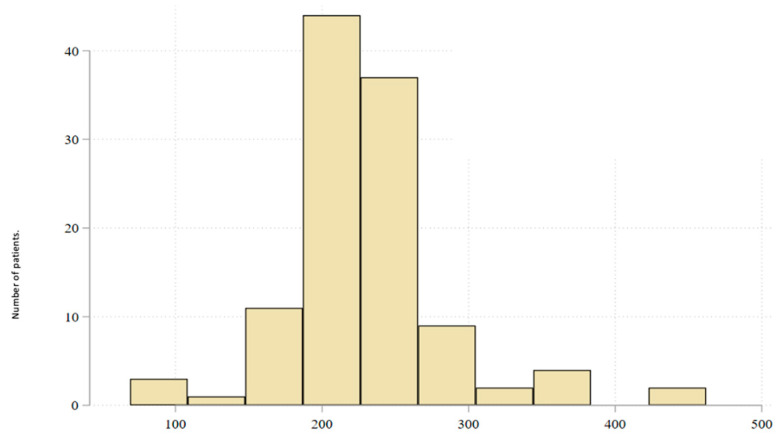
Time from multidisciplinary tumor board (MDT) decision to surgery (in days).

All patients received neoadjuvant endocrine therapy with oral letrozole at a standard dose of 2.5 mg daily. Treatment continued until surgery with a median duration of 225 days. The longest treatment period was 462 days, attributed to the patient’s personal decision to delay hospitalization and surgery due to concerns related to the COVID-19 pandemic (Figure 2). During treatment, patients attended follow-up visits at the oncology outpatient clinic every 2 to 3 months for physical examinations and additional assessments, following institutional guidelines.

A subgroup of 80 patients (Group B) underwent an interim core needle biopsy after approximately 4 weeks of therapy (median 29 days; 14–122 days). In two patients, the interim tumor biopsy was performed more than 80 days after the initiation of therapy due to challenges related to the epidemiological situation in the country at that time. This procedure was performed during standard clip marker placement and did not require any additional intervention. The remaining 47 patients (Group A) continued treatment without re-biopsy. Upon completion of the preoperative endocrine therapy, all patients were re-evaluated by a multidisciplinary tumor board (MDT) and referred for definitive breast surgery.

### 2.3. Pathological and Biomarker Assessment

Histopathological and immunohistochemical (IHC) evaluations were performed on tumor specimens obtained at three time points: baseline (diagnostic core biopsy), interim biopsy (Group B only) and surgical resection specimens. The analysis included tumor type and grade, estrogen receptor (ER) and progesterone receptor (PgR) expression, *HER2* status, and *Ki-67* proliferation index. Standardized staining protocols and institutional scoring methods were used throughout.

ER and PgR positivity were defined as nuclear staining in ≥1% of tumor cells. *HER2* status was assessed using immunohistochemistry (IHC) with the **VENTANA PATHWAY anti-HER2/neu (clone 4B5)** assay (Ventana Medical Systems, Tucson, AZ, USA). A standard four-tier scoring system was used (0, 1+, 2+, 3+) according to current ASCO/CAP guidelines. In cases with equivocal HER2 IHC results (2+), additional fluorescence in situ hybridization (FISH) was performed using the Vysis HER2/CEP17 probe (Abbott Molecular Inc., Des Plaines, IL, USA)to determine *HER2* gene amplification status. Only patients with confirmed *HER2-negative* tumors (IHC 0/1+ or IHC 2+ with FISH-negative) at baseline and interim biopsy were included in the final analysis. The *Ki-67* index was reported as the percentage of positively stained invasive tumor cells among at least 500 counted cells. Tumor response was classified using the Residual Cancer Burden (RCB) system, and pathological complete response (pCR) was defined as the absence of invasive cancer in the breast and lymph nodes (ypT0/is, ypN0).

### 2.4. Statistical Analysis

Descriptive statistics were used to summarize baseline characteristics. Continuous variables were reported as means with standard deviations (SDs) or as medians with interquartile ranges (IQRs), depending on the distribution. Categorical variables were presented as frequencies and percentages. To compare categorical variables between the interim biopsy group and the non-biopsy group, chi-square or Fisher’s exact tests were used, as appropriate. For comparisons of continuous variables between groups, the Wilcoxon rank-sum test was applied. The Wilcoxon signed-rank test was used to assess changes in continuous variables over time within the same patients.

To evaluate the predictive value of post-treatment *Ki-67* levels (<2.7% vs. ≥2.7%) based on early biomarker changes, we performed a Classification and Regression Tree (CART) analysis using the R package *rpart*. Predictor variables included baseline and interim *Ki-67* values, the absolute change in *Ki-67, PgR* expression, and histological grade at baseline and interim biopsy. Estrogen receptor (ER) expression was excluded due to minimal variability across the cohort.

All statistical analyses were performed using STATA/SE 17 (StataCorp LLC, College Station, TX, USA) and R version 4.1.0 (The R Foundation for Statistical Computing, Vienna, Austria). A *p*-value of <0.05 was considered statistically significant.

## 3. Results

### 3.1. Baseline Characteristics of the Study Population

A total of 127 postmenopausal patients with *HR+/HER2−* breast cancer were included in the study. The median age was 67 years, and the mean duration of neoadjuvant endocrine therapy (NET) was 7.7 months. Most tumors (53.2%) were clinically staged as cT2 at diagnosis. The mean baseline tumor size was 41.1 mm in Group A and 37.0 mm in Group B (*p* = 0.510). Lymph node involvement at diagnosis was significantly more frequent in Group B compared to Group A (58.8% vs. 34.0%, *p* = 0.026). In terms of histological type, 80.3% of tumors were classified as invasive ductal carcinoma of no special type (NST). Grade 2 (G2) tumors predominated in both groups, with 83 patients (67.7%). There was no significant difference in grade distribution between groups (*p* = 0.146). Letrozole was used in 124 of the 127 patients. All patients were ER-positive, with a mean *ER* expression of 97.6%, and PgR-positive, with a baseline mean *PgR* expression of 57.5%. Initial *Ki-67* levels were <27% in 102 patients (80.3%). All patients had *HER2*-negative tumors at baseline (IHC 0/1+ or 2+ with negative FISH). At diagnosis, 64 patients (50.4%) were qualified for mastectomy and 63 (49.6%) for breast-conserving surgery (BCS) (*p* = 0.145) Table 1.

### 3.2. Interim Biopsy During Neoadjuvant Endocrine Therapy

**Figure 3:**
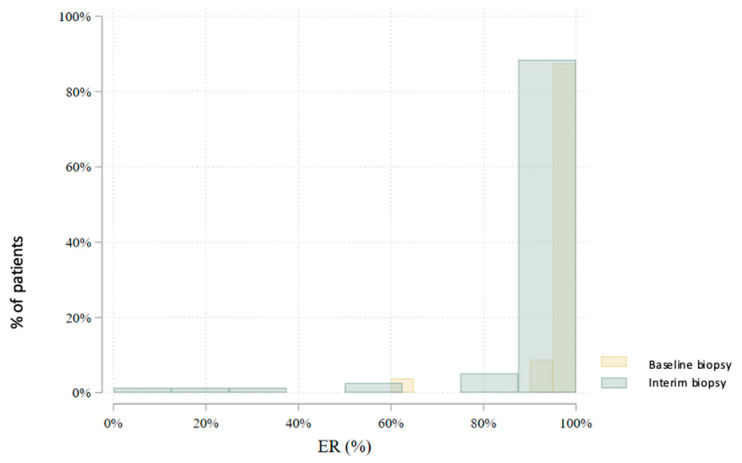
Comparison of estrogen receptor (ER) expression levels between the baseline and interim core biopsies (*p* = 0.004).

**Figure 4:**
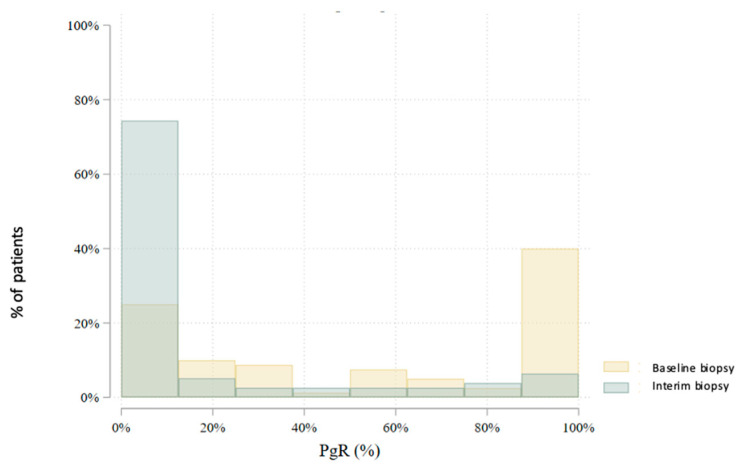
Comparison of estrogen receptor (PgR) expression levels between the baseline and interim core biopsies (*p* < 0.0001).

**Figure 5:**
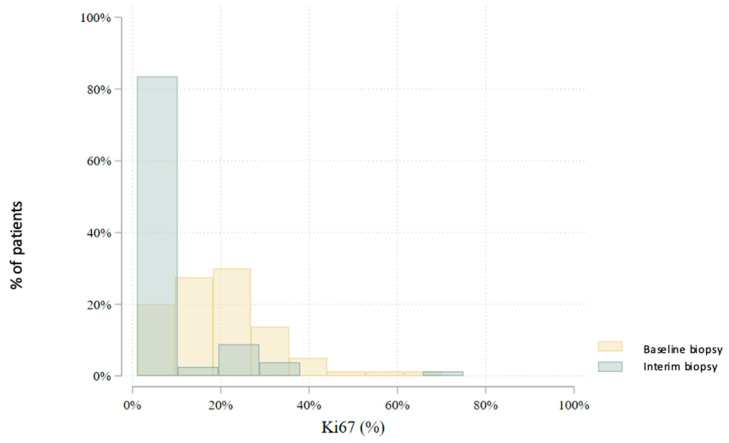
Comparison of Ki67 levels between the initial and interim biopsy (*p* < 0.0001).

In total, 80 patients underwent an interim core needle biopsy approximately four weeks after NET initiation. Two patients (2.5%) were excluded from further analysis: one due to non-diagnostic biopsy and another due to radiological complete response. Thus, the interim biopsy analysis includes 78 patients. Histological subtype analysis showed a slight increase in lobular carcinomas (from 22.5% to 28.2%), with six subtype conversions observed. Histological grade changed in 47.4% of patients: it decreased in 41.0%, increased in 6.4%, and remained stable in 52.6%. No grade 3 tumors were found in the interim biopsy. Estrogen receptor (ER) positivity was preserved in all but one patient (98.7%) (Figure 3). This patient, with a strong clinical response and preserved progesterone receptor (PgR) expression, continued treatment and showed ER positivity postoperatively. PgR expression declined notably during NET, with 79.5% of patients having ≤20% PgR expression in the interim biopsy (Figure 4). *HER2* status conversion was detected in five patients (6.4%) in the interim biopsy, leading to exclusion from further analysis due to systemic therapy modification. These cases had initial *HER2* scores of 1+ or 2+ (FISH-negative), with subsequent IHC 3+ or FISH amplification. The *Ki-67* index decreased significantly during NET. In the first biopsy, 22.5% had *Ki-67* ≥ 27%, whereas only 5.1% retained this level in the interim biopsy (Figure 5). Notably, 33.3% achieved *Ki-67* < 2.7%. Detailed comparison of ER, PgR, and *Ki-67* expression between initial and interim biopsies showed statistically significant declines in PgR and *Ki-67* (*p* < 0.0001) and a smaller but significant reduction in ER (*p* = 0.004) (Table 2).

### 3.3. Postoperative Results

Surgical treatment was performed in 120 out of 127 patients originally included in the study. Five patients (3.9%) declined surgery and two (1.6%) developed distant metastases during neoadjuvant endocrine therapy (NET), leading to the withdrawal of surgical intervention. Additionally, five patients were excluded from the analysis due to *HER2* overexpression detected in the control biopsy. Thus, postoperative histopathological analysis included 115 patients. A pathological complete response (pCR) was observed in four patients (3.5%). Breast-conserving surgery (BCS) was performed in 60 patients (52.2%), including 47 (78.3%) who underwent sentinel lymph node biopsy (SLNB) and 13 (21.7%) who required axillary lymph node dissection (ALND). ALND was not performed in 75 patients (65.7%): in 28 (37.3%) undergoing mastectomy and 47 (62.7%) undergoing BCS. No statistically significant difference in surgical approach was observed between study groups (*p* = 0.317). In 79 patients (68.7%), the surgical procedure was consistent with the initial pre-NET surgical plan. Less extensive surgery was performed in 24 patients (14.3%), most often due to tumor regression during NET. Notably, patients initially qualified for radical mastectomy were the most likely to forgo surgery (Table 3).

Residual cancer burden (RCB) was evaluated in all 115 operated patients. RCB-0 (pCR) was observed in 4 (3.5%) cases, RCB-I in 18 (15.7%), RCB-II in 64 (55.6%), and RCB-III in 29 (25.2%). No statistically significant differences were found between groups (*p* = 0.125).

**Figure 6:**
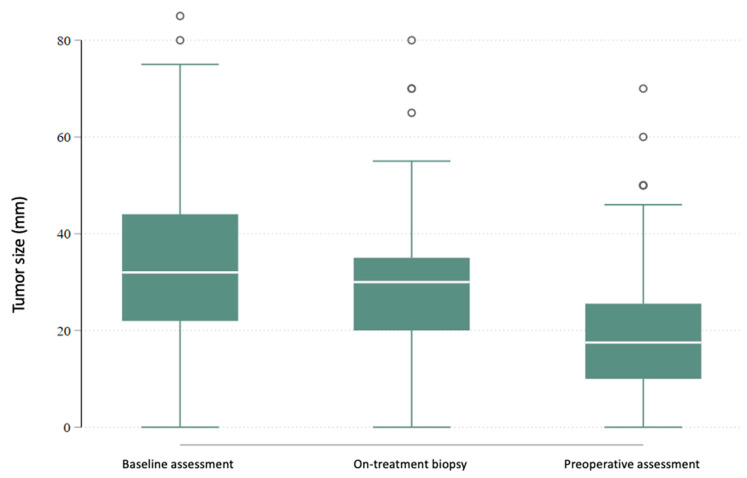
Tumor size (mm) at baseline, during treatment (~4 weeks), and preoperatively following neoadjuvant endocrine therapy (NET). Median tumor size decreased from 32.0 mm to 17.0 mm, corresponding to a median relative reduction of 47.0% (*p* < 0.0001).

Tumor size in the postoperative pathology reports was most commonly classified as pT1 (47.8%). The mean tumor size was 22.6 mm in group A and 23.4 mm in group B (*p* = 0.952), with a median of 19 mm in both groups. Imaging and physical examination conducted before and after NET showed a significant reduction in tumor size (*p* < 0.0001), which often led to downstaging and less extensive surgery (Figure 6).

Nodal involvement was confirmed in 57 patients (49.6%). The most frequent nodal stage was pN1 (26.1%). No lymph node metastases (pN0) were observed in 58 patients (50.4%), with a higher rate of pN0 in group B compared to group A (52.9% vs. 46.8%, *p* = 0.015).

**Figure 7:**
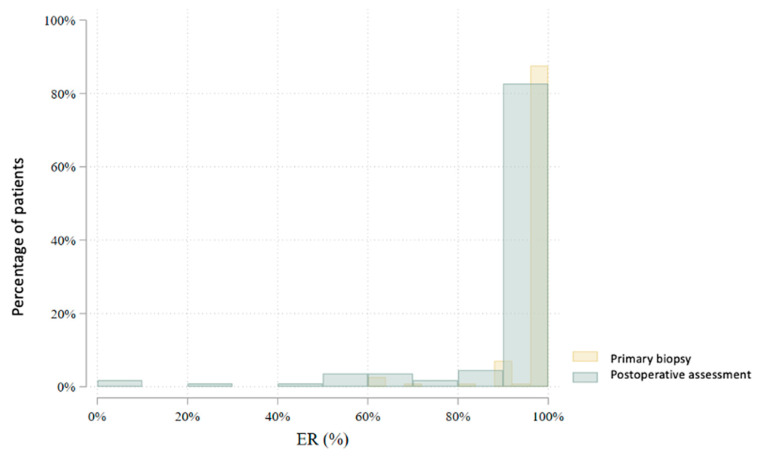
Comparison of estrogen receptor (ER) expression levels between the primary biopsy and the postoperative assessment (*p* < 0.0001).

**Figure 8:**
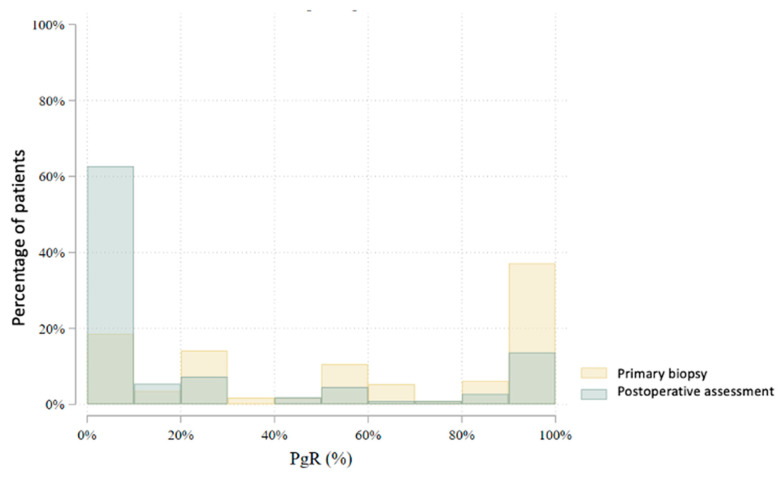
Comparison of PgR receptor levels between the initial biopsy and the postoperative assessment (*p* < 0.0001).

**Figure 9:**
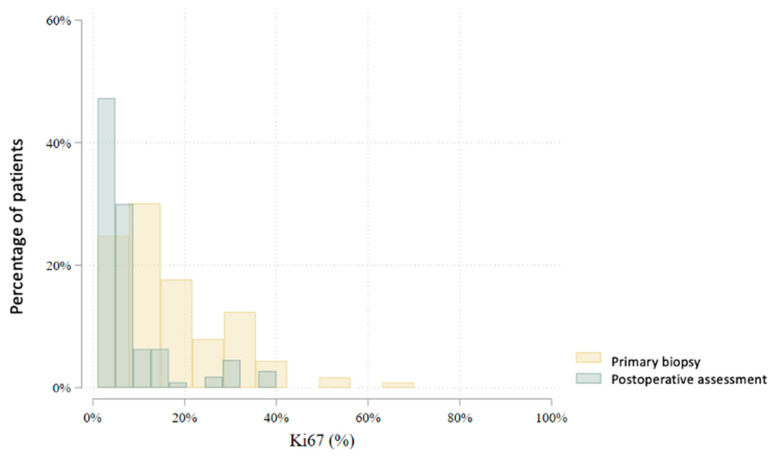
Comparison of Ki67 levels between the initial biopsy and the postoperative assessment (*p* < 0.0001).

According to the AJCC/UICC 8th edition staging system, most patients were classified as stage II (46.9%) after NET. Stage I was noted in 33.3% and stage III in 19.8%. Compared to baseline clinical staging, there was a notable shift: the proportion of patients in stage I increased (from 3.2% to 33.3%), while stage II and III proportions decreased. In the postoperative histopathological evaluation, invasive ductal carcinoma (no special type, NST) was the predominant subtype (65.8%), followed by invasive lobular carcinoma (26.1%) and other histologies (8.1%). Compared to initial biopsy results, there was an increase in the proportion of lobular carcinomas and the identification of rare histological subtypes not initially reported. The histological grade distribution also changed postoperatively. The frequency of grade 1 tumors increased from 25.2% to 48.2%, while grade 2 and 3 tumors decreased from 67.7% to 47.3% and from 7.1% to 4.5%, respectively. In group B, grade 3 tumors were observed in 7.7%, while no such tumors were found in group A. Estrogen receptor (ER) expression was present in 109 (98.2%) of 111 evaluable tumors in postoperative specimens Figure 7. Progesterone receptor (PgR) expression > 20% was observed in 28.8% of tumors, significantly more frequently in group A than in group B (42.2% vs. 19.7%, *p* = 0.010) (Figure 8). Mean ER expression was 95.2% in group A and 88.0% in group B (*p* = 0.239). A *Ki67* index <27% was observed in 92.8% of patients, while 37.8% had a very low *Ki67* index <2.7%. A significantly higher proportion of very low *Ki67* was observed in group B compared to group A (47.0% vs. 24.4%, *p* = 0.016), although the difference in mean *Ki67* was not statistically significant (7.6% vs. 6.3%, *p* = 0.094) (Figure 9).

**Figure 10:**
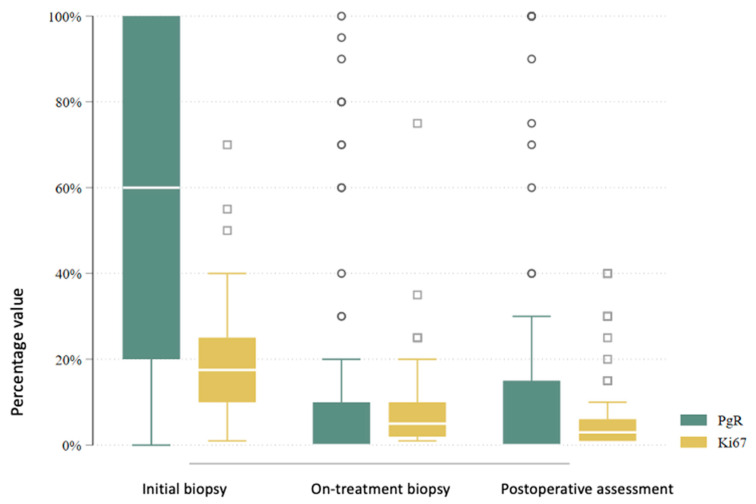
Comparison of Ki67 and PgR levels in the initial biopsy, control biopsy, and postoperative assessment.

When comparing receptor expression and *Ki67* values between the initial biopsy and postoperative specimens, a statistically significant decrease was observed for all three biomarkers (ER, PgR, and *Ki67*; *p* < 0.0001 for all) (Table 4). A simultaneous reduction in both *Ki67* and PgR was observed in 67.5% of patients, while only 1.8% demonstrated increases in both (Figure 10).

### 3.4. Analysis of Ki-67 Trajectories in Response to NET

**Figure 11:**
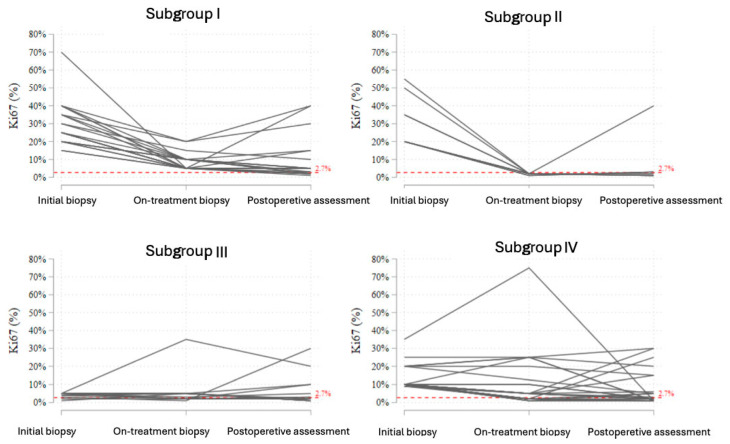
Trajectories of Ki-67 expression across three timepoints (initial biopsy, on-treatment biopsy, and postoperative assessment) in patients undergoing neoadjuvant endocrine therapy, stratified into four subgroups based on baseline and on-treatment Ki-67 dynamics. The red dashed line indicates the 2.7% threshold. Subgroup I: initial Ki-67 > 2.7% with partial decrease but no drop below 2.7%; Subgroup II: initial Ki-67 > 2.7% with decrease below 2.7% during treatment; Subgroup III: initial Ki-67 < 10%, mostly stable or increasing during treatment; Subgroup IV: initial Ki-67 ≥ 10% with no significant decrease during treatment.

To further evaluate the dynamic changes in tumor proliferation during neoadjuvant endocrine therapy (NET), individual *Ki-67* trajectories were analyzed in a subgroup of patients who underwent an on-treatment biopsy. Patients were categorized into four subgroups based on initial and on-treatment Ki-67 values (Figure 11).

• **Subgroup I** included patients with initially elevated Ki-67 who showed a reduction during treatment, but the on-treatment value remained ≥2.7%. In this subgroup, most patients experienced further stabilization or a modest decline postoperatively, although complete suppression of proliferation (<2.7%) was not achieved.
• **Subgroup II** consisted of patients who showed a profound early decline in Ki-67 to <2.7% at the on-treatment biopsy. In nearly all cases, this suppression persisted in the postoperative samples, reflecting a strong endocrine response and sustained biological effect.
• **Subgroup III** comprised patients with initially low Ki-67 (<10%), who demonstrated stable or slightly increased values during treatment. The majority remained within the low-proliferative range throughout therapy.
• **Subgroup IV** represented patients with elevated baseline Ki-67 (≥10%) who did not achieve significant suppression during treatment. Most of these tumors maintained a Ki-67 above 2.7%, although isolated cases exhibited paradoxical fluctuations or late declines postoperatively.

This trajectory-based classification highlights the heterogeneity of biological responses to NET and supports the potential utility of on-treatment Ki-67 evaluation for early identification of endocrine-sensitive tumors.

## 4. Discussion

Endocrine therapy remains a cornerstone in the management of hormone receptor-positive breast cancer across both early and advanced disease settings. In postmenopausal women, aromatase inhibitors (AIs) have demonstrated superior efficacy compared to tamoxifen in both the neoadjuvant and adjuvant settings. In the metastatic context AIs are increasingly combined with targeted therapies such as CDK4/6 or mTOR inhibitors to enhance treatment outcomes [9]. The predictive and prognostic value of biomarkers—including estrogen receptor (ER), progesterone receptor (PgR), and *Ki-67*—has been explored in multiple clinical trials employing various endocrine agents [10]. Despite the advantages of aromatase inhibitors highlighted in landmark neoadjuvant trials such as P024 and Z1031A, the uptake of NET in clinical practice remains modest. Our study enrolled only postmenopausal women, in line with current recommendations and available evidence regarding the use of neoadjuvant endocrine therapy (NET) [11]. Hormone receptor-positive (HR+) breast cancer is more common in this population. The median age in our cohort was 67 years (mean: 67.3), with the largest subgroup (50.4%) being 60–69 years of age. This reflects the epidemiology of breast cancer in developed countries, where the peak incidence occurs before age 70 [12]. Women under 60 accounted for 12.6% of our cohort, while patients aged ≥80 years represented only 7.1%. Only female patients were enrolled, given the extremely low incidence of male breast cancer (0.1–6.5 cases per 100,000) [13]. There were no statistically significant differences in age distribution between patients undergoing re-biopsy and those without it (*p* = 0.734). The age profile of our population is comparable to other pivotal studies on neoadjuvant endocrine therapy. For example, in the IMPACT trial, which evaluated preoperative anastrozole, tamoxifen, or a combination, the median age ranged from 71.5 to 73.2 years depending on the treatment arm [14]. In the ACOSOG Z1031 trial, which focused on preoperative aromatase inhibitor therapy, the median age varied between 60 and 66 years across subgroups [6]. Similarly, in the randomized PROACT study, where only postmenopausal women were included, the mean age was 67.3 years in the anastrozole group and 66.7 in the tamoxifen arm [15]. The present prospective study analyzed clinical and histopathological responses to NET in a cohort of 127 postmenopausal women with HR+/HER2− early breast cancer treated at a single high-volume oncology center. Special emphasis was placed on the evaluation of *Ki-67* and PgR as early predictive markers of therapeutic response. Our study confirmed a statistically significant reduction in tumor size (median reduction of 47%, *p* < 0.0001), accompanied by a shift in surgical eligibility, with 16.2% of patients initially scheduled for mastectomy ultimately undergoing breast-conserving procedures. These findings are consistent with the original goals of NET, namely tumor downstaging and the facilitation of less extensive surgery, particularly in elderly populations [10,16,17]. The median duration of treatment in our cohort was 7.7 months, aligning with international recommendations that suggest a minimum of 3–4 months, with optimal duration extending up to 6–12 months depending on tumor dynamics and clinical response. Short-term use of NET (2–4 weeks) prior to surgery and monitoring changes in the *Ki-67* index have been recommended as a method for assessing tumor sensitivity to endocrine therapy [18,19,20]. This approach aims to identify patients who may safely avoid chemotherapy due to an effective endocrine response. The most recent recommendations from the 2023 St. Gallen International Consensus further emphasized the role of individualized treatment strategies in early HR+/HER2− breast cancer and supported the use of dynamic biomarker changes, such as *Ki-67*, to guide clinical decision-making. This supports the utility of NET as a viable de-escalation strategy for systemic and surgical treatment in appropriately selected patients [21]. From a biological perspective, the majority of tumors exhibited a decrease in *Ki-67* expression during NET. While baseline *Ki-67* values were <27% in 80.3% of tumors, a decline to <2.7% was observed in 33.3% of patients. A sustained reduction in *Ki-67* was most pronounced in a specific subgroup (Group II), defined by an early drop below 2.7% in the control biopsy. This group maintained low proliferation rates in postoperative specimens, suggesting that early *Ki-67* suppression may serve as a robust surrogate of long-term endocrine sensitivity. Our results confirm prior findings from the POETIC and IMPACT trials, which emphasized the prognostic significance of on-treatment *Ki-67* levels and their potential role in guiding adjuvant systemic therapy decisions [14,22]. Simultaneous reductions in both *Ki-67* and PgR were observed in 67% of patients, while only 1.8% demonstrated a dual increase. Although the biological underpinnings of parallel declines in *Ki-67* and PgR remain unclear, this pattern may reflect a deeper suppression of estrogen signaling pathways under AI therapy. Interestingly, PgR expression dropped markedly from a baseline mean of 57.5% to 16.4% at interim biopsy and 22.9% in the surgical specimen (*p* < 0.0001), a phenomenon also reported by Covadonga et al. in their 2022 study [23]. The clinical utility of PgR dynamics during NET remains a subject of ongoing research. Histological analysis confirmed that tumor grade decreased significantly during therapy. Among patients with baseline grade 3 (G3) tumors, 25% demonstrated regression to grade 1 (G1) and 75% to grade 2 (G2) following NET. Furthermore, no G3 tumors were observed in the interim biopsies, and overall, 41% of cases showed histological downgrading during treatment. These findings support the notion that NET is effective even in patients with high-grade tumors, challenging the historical hesitation to recommend endocrine therapy in such cases. Our observations are consistent with the data reported by Covadonga et al., in which NET led to significant histological-grade reductions after 4 weeks of therapy, although G3 tumors re-emerged postoperatively in a minority of patients. This phenomenon may reflect partial biological suppression of tumor aggressiveness under NET, particularly in terms of proliferation, but may also be explained by tumor heterogeneity and sampling variability. As core biopsies assess only a portion of the tumor mass, changes in grade should be interpreted with caution, and final histopathological evaluation remains essential for guiding adjuvant treatment. *HER2* receptor status conversion was identified in 6.3% of patients (5 during interim biopsy, 3 in postoperative specimens) a phenomenon increasingly recognized in the context of systemic therapy. As *HER2*-positive disease mandates an entirely different systemic treatment approach, including anti-HER2 therapy, these findings underscore the necessity of reassessing receptor status either intra-treatment or postoperatively, especially in the setting of unexpected progression or histological evolution [24]. Surgical outcomes further support the clinical utility of NET [10]. Of the 127 patients, 52.2% underwent breast-conserving surgery (BCS) and 16.2% experienced conversion from initial mastectomy eligibility. While the rate of surgical de-escalation may appear modest, it aligns with reported conversion rates from studies such as Z1031 and P024. Sentinel lymph node biopsy was performed in 65.2% of cases. Importantly, the nodal pathological status after NET showed that 50.4% of patients were ypN0, consistent with data suggesting that NET may also impact axillary disease in selected cases, albeit less frequently than neoadjuvant chemotherapy [25,26]. Pathologic complete response (pCR) was achieved in 3.5% of patients, an expectedly low rate given the intrinsic endocrine responsiveness but modest chemosensitivity of luminal tumors. Residual Cancer Burden (RCB) classification revealed that 71.3% of patients achieved RCB I/II, indicating partial or moderate residual disease, while 25.2% had RCB III, indicative of endocrine resistance. These proportions are consistent with those reported in NET trials such as ABCSG-34 and CORALLEEN [27,28]. Although RCB is traditionally used in the context of chemotherapy, its utility in endocrine-based strategies is emerging, particularly as a surrogate for long-term outcome prediction and a potential stratifier for post-NET treatment intensification [29]. In conclusion, our findings confirm that NET with letrozole is a clinically and biologically effective strategy for tumor downstaging and biological modulation in postmenopausal patients with HR+/HER2− breast cancer. The early decline in *Ki-67* and PgR expression provides valuable insights into endocrine responsiveness, while interim biopsy enables real-time treatment monitoring. Our analysis of serial *Ki-67* assessments across three timepoints also underscores the value of interim biopsy as a tool for monitoring endocrine response. Patients who achieved *Ki-67* suppression below the 2.7% threshold at week 4 maintained this favorable profile in most cases, as reflected in postoperative specimens. This suggests that early *Ki-67* decline not only captures initial endocrine sensitivity but may also predict long-term suppression of tumor proliferation. Moreover, response trajectory analysis revealed biologically distinct subgroups with potential prognostic relevance. These findings support the growing evidence for dynamic biomarker monitoring during NET and may aid in identifying patients who require treatment escalation or alternative strategies. This study has several limitations. The single-center design and relatively small sample size may limit the generalizability of the findings, particularly in subgroup analyses (e.g., patients with post-treatment *Ki-67* < 2.7%), where statistical power is reduced. Furthermore, biopsy timing and treatment duration were not standardized, potentially introducing heterogeneity in treatment response. Although cN status was unevenly distributed between groups, it did not appear to significantly influence histological response or *Ki-67* dynamics in our cohort. Nevertheless, this imbalance should be considered when interpreting the subgroup analyses. As the study was not designed to assess the impact of NET duration on radiologic or pathologic outcomes, no formal correlation analysis was performed. Several patients were excluded from the final analysis at different stages. Surgery was not performed in seven patients (5.5%)—five declined surgery and two developed distant metastases during NET. An additional five patients were excluded due to HER2 overexpression detected in the second biopsy, resulting in systemic treatment modification. These exclusions were based on predefined, clinically justified criteria to ensure biological consistency and the validity of endocrine response assessment. Given their small number and clear rationale, they are unlikely to introduce significant bias. Among the key strengths of this study are the prospective design, homogeneous patient population, and detailed pathological evaluation. Importantly, the study reflects real-world clinical practice in a tertiary oncology center, offering insights relevant to everyday management of postmenopausal women with HR+/HER2− early breast cancer.

## 5. Conclusions

This prospective study confirms that neoadjuvant endocrine therapy (NET) with letrozole is a clinically effective and biologically meaningful strategy for tumor downstaging in postmenopausal patients with HR+/HER2− early breast cancer. The observed early decline in *Ki-67* and PgR expression provides valuable insight into tumor endocrine sensitivity and may serve as a predictive tool for monitoring therapeutic response. Interim core needle biopsy after four weeks of NET allowed for real-time evaluation of biomarker dynamics, identifying distinct biological response patterns. Although the study has limitations, including its single-center design and lack of standardized treatment duration, the findings support the utility of serial biomarker assessment to guide individualized treatment decisions. Further multicenter studies are needed to validate these observations and determine the long-term prognostic significance of early biological changes during NET.

## Figures and Tables

**Table 1:** Baseline clinical and pathological characteristics of patients with and without interim biopsy (Group B and Group A, respectively; N = 127). T—tumor; N—nodes; ER—estrogen receptor; PGR—progesterone receptor; MRM—modified radical mastectomy; MST—mastectomy; SLNB—sentinel lymph node biopsy; ALND—axillary lymph node dissection.

Clinical Characteristics at Baseline		Group A (No Interim Biopsy)	Group B (with Interim Biopsy)	Number of Patients	*p*-Value
Age	50–59	5 (10.6%)	11 (13.8%)	16 (12.6%)	0.734
	60–69	26 (55.3%)	38 (47.5%)	64 (50.4%)	
	70–79	12 (25.5%)	26 (32.5%)	38 (29.9%)	
	80+	4 (8.5%)	5 (6.3%)	9 (7.1%)	
	Total	47 (100.0%)	80 (100.0%)	127 (100.0%)	
cT	T1	4 (8.5%)	5 (6.3%)	9 (7.1%)	0.738
	T2	22 (46.8%)	45 (57.0%)	67 (53.2%)	
	T3	17 (36.2%)	23 (29.1%)	40 (31.7%)	
	T4	4 (8.5%)	6 (7.6%)	10 (7.9%)	
	Total	47 (100.0%)	79 (100.0%)	126 (100.0%)	
cN	N0	31 (66.0%)	33 (41.3%)	64 (50.4%)	0.026
	N1	11 (23.4%)	34 (42.5%)	45 (35.4%)	
	N2	5 (10.6%)	13 (16.3%)	18 (14.2%)	
	Total	47 (100.0%)	80 (100.0%)	127 (100.0%)	
Histological subtype of the primary tumor	Lobular	7 (14.9%)	18 (22.5%)	25 (19.7%)	0.298
	NOS	40 (85.1%)	62 (77.5%)	102 (80.3%)	
	Total	47 (100.0%)	80 (100.0%)	127 (100.0%)	
Histological grade (G)	G1	15 (31.9%)	17 (21.3%)	32 (25.2%)	0.138
	G2	31 (66.0%)	55 (68.8%)	86 (67.7%)	
	G3	1 (2.1%)	8 (10.0%)	9 (7.1%)	
	Total	47 (100.0%)	80 (100.0%)	127 (100.0%)	
ER	Yes	47 (100.0%)	80 (100.0%)	127 (100.0%)	
	Total	47 (100.0%)	80 (100.0%)	127 (100.0%)	
PGR	<=20%	11 (23.4%)	28 (35.0%)	39 (30.7%)	0.171
	>20%	36 (76.6%)	52 (65.0%)	88 (69.3%)	
	Total	47 (100.0%)	80 (100.0%)	127 (100.0%)	
HER2	No	47 (100.0%)	80 (100.0%)	127 (100.0%)	
	Total	47 (100.0%)	80 (100.0%)	127 (100.0%)	
Ki67 (%)	<=20%	36 (76.6%)	55 (68.8%)	91 (71.7%)	0.344
	>20%	11 (23.4%)	25 (31.3%)	36 (28.3%)	
	Total	47 (100.0%)	80 (100.0%)	127 (100.0%)	
Ki67 (%)	<2.7%	1 (2.1%)	2 (2.5%)	3 (2.4%)	0.894
	>=2.7%	46 (97.9%)	78 (97.5%)	124 (97.6%)	
	Total	47 (100.0%)	80 (100.0%)	127 (100.0%)	
Drug	Letrozole	47 (100.0%)	77 (96.3%)	124 (97.6%)	0.406
	Anastrozole	0 (0.0%)	1 (1.3%)	1 (0.8%)	
	Tamoxifen	0 (0.0%)	2 (2.5%)	2 (1.6%)	
	Total	47 (100.0%)	80 (100.0%)	127 (100.0%)	
Primary surgical qualification	MRM	9 (19.1%)	29 (36.3%)	38 (29.9%)	0.145
	MST+SLNB	13 (27.7%)	13 (16.3%)	26 (20.5%)	
	BCS/TU+SLNB	21 (44.7%)	34 (42.5%)	55 (43.3%)	
	BCS/TU+ALND	4 (8.5%)	4 (5.0%)	8 (6.3%)	
	Total	47 (100.0%)	80 (100.0%)	127 (100.0%)	

**Table 2:** Comparison of ER, PgR, and Ki67 levels between the initial and interim biopsy in the interim biopsy group (N = 78).

	Baseline Biopsy				Interim Biopsy				*p*-Value
	N	Mean (SD)	Median (IQR)	Range	N	Mean (SD)	Median (IQR)	Range	
Ki67 (%)	80	19.1 (13.3)	20.0 (10.0–25.0)	1–70	78	8.0 (10.8)	5.0 (2.0–10.0)	1–75	0.000
ER (%)	80	97.7 (8.0)	100.0 (100.0–100.0)	60–100	78	93.8 (18.2)	100.0 (100.0–100.0)	0–100	0.004
PgR (%)	80	55.5 (40.8)	60.0 (15.0–100.0)	0–100	78	16.4 (29.2)	0.0 (0.0–15.0)	0–100	0.000

**Table 3:** Surgical procedures performed compared to initial surgical qualification (N = 120).

Surgical Procedure Performed	Initial Qualification				Total
N (%)	MRM	MST+SLNB	BCS/TU+SLNB	BCS/TU+ALND	
No surgery performed	5 (14.3%)	1 (3.8%)	1 (2.0%)	0 (0.0%)	7 (5.8%)
MRM	15 (42.9%)	2 (7.7%)	0 (0.0%)	0 (0.0%)	17 (14.2%)
MST+SLNB	5 (14.3%)	18 (69.2%)	5 (9.8%)	0 (0.0%)	28 (23.3%)
MST+ALND	5 (14.3%)	1 (3.8%)	0 (0.0%)	1 (12.5%)	7 (5.8%)
BCS/TU+SLNB	2 (5.7%)	3 (11.5%)	38 (74.5%)	3 (37.5%)	46 (38.3%)
BCS/TU+ALND	3 (8.6%)	0 (0.0%)	7 (13.7%)	3 (37.5%)	13 (10.8%)
Other	0 (0.0%)	1 (3.8%)	0 (0.0%)	1 (12.5%)	2 (1.7%)
Total	35 (100.0%)	26 (100.0%)	51 (100.0%)	8 (100.0%)	120 (100.0%)

**Table 4:** Comparison of ER, PgR, and Ki67 levels between the initial biopsy and the postoperative assessment in the group of patients who underwent surgery (N = 115).

	Baseline Biopsy				Postoperative Assessment				*p*-Value
	N	Mean (SD)	Mediana (IQR)	Range	N	Mean (SD)	Mediana (IQR)	Range	
Ki67 (%)	115	17.0 (12.7)	10.0 (9.0–25.0)	1–70	111	7.1 (9.0)	5.0 (2.0–6.0)	1–40	0.000
ER (%)	115	97.8 (7.5)	100.0 (100.0–100.0)	60–100	111	90.9 (18.5)	100.0 (90.0–100.0)	0–100	0.000
PgR (%)	115	57.5 (38.7)	60.0 (20.0–100.0)	0–100	111	22.9 (35.9)	0.0 (0.0–30.0)	0–100	0.000

## Data Availability

The data supporting the findings of this study are available from the corresponding author upon reasonable request. Due to ethical and legal restrictions, individual patient data are not publicly available.
